# Vestibular-evoked myogenic potential triggered by galvanic vestibular stimulation may reveal subclinical alterations in human T-cell lymphotropic virus type 1-associated myelopathy

**DOI:** 10.1371/journal.pone.0200536

**Published:** 2018-07-12

**Authors:** Ludimila Labanca, Júlia Fonseca de Morais Caporali, Sirley Alves da Silva Carvalho, José Roberto Lambertucci, Anna Bárbara de Freitas Carneiro Proietti, Luiz Cláudio Ferreira Romanelli, Paul Avan, Fabrice Giraudet, Bárbara Oliveira Souza, Kyonis Rodrigues Florentino, Denise Utsch Gonçalves

**Affiliations:** 1 Programa de Pós-Graduação em Infectologia e Medicina Tropical, Faculdade de Medicina da Universidade Federal de Minas Gerais, Belo Horizonte, Minas Gerais, Brazil; 2 Programa de Pós-Graduação em Ciências Fonoaudiológicas, Faculdade de Medicina da Universidade Federal de Minas Gerais, Belo Horizonte, Minas Gerais, Brazil; 3 Fundação Centro de Hematologia e Hemoterapia de Minas Gerais (HEMOMINAS), Belo Horizonte, Minas Gerais, Brazil; 4 Laboratoire de Biophysique Neurosensorielle, Faculté de Médecine, Université Clermont Auvergne, Clermont Ferrand, Auvergne, France; Universidade Estadual de Ciencias da Saude de Alagoas, BRAZIL

## Abstract

**Background:**

Vestibular-evoked myogenic potential triggered by galvanic vestibular stimulation (galvanic-VEMP) evaluates the motor spinal cord and identifies subclinical myelopathies. We used galvanic-VEMP to compare spinal cord function in individuals infected with human T-cell lymphotropic virus type 1 (HTLV-1) from asymptomatic status to HTLV-1-associated myelopathy (HAM).

**Methodology/Principal findings:**

This cross-sectional study with 122 individuals included 26 HTLV-1-asymptomatic carriers, 26 individuals with possible HAM, 25 individuals with HAM, and 45 HTLV-1-seronegative individuals (controls). The groups were similar regarding gender, age, and height. Galvanic stimuli (duration: 400 ms; intensity: 2 mA) were applied bilaterally to the mastoid processes and VEMP was recorded from the gastrocnemius muscle. The electromyographic parameters investigated were the latency and amplitude of the short-latency (SL) and medium-latency (ML) responses. While SL and ML amplitudes were similar between groups, SL and ML latencies were delayed in the HTLV-1 groups compared to the control group (p<0.001). Using neurological examination as the gold standard, ROC curve showed an area under the curve of 0.83 (p<0.001) for SL and 0.86 (p<0.001) for ML to detect spinal cord injury. Sensibility and specificity were, respectively, 76% and 86% for SL and 79% and 85% for ML. Galvanic-VEMP disclosed alterations that were progressive in HTLV-1-neurological disease, ranging from SL delayed latency in HTLV-1-asymptomatic carriers, SL and ML delayed latency in possible HAM group, to absence of VEMP response in HAM group.

**Conclusions/Significance:**

The worse the galvanic-VEMP response, the more severe the myelopathy. Galvanic-VEMP alteration followed a pattern of alteration and may be a prognostic marker of progression from HTLV-1-asymptomatic carrier to HAM.

## Introduction

Human T-cell lymphotropic virus type 1 (HTLV-1)-associated myelopathy (HAM) is a chronic inflammatory disease that occurs in approximately 3% of HTLV-1-infected individuals [1,2]. HAM affects mainly the lower thoracic spine [3–5], which leads to progressive postural and gait impairments [6].

The diagnosis of HAM is based on clinical and imaging methods [7,8]. During the early stages of HAM, magnetic resonance imaging (MRI) cannot detect alterations in the patients, even in those with signs and symptoms of upper motor neuron disease [9,10]. Thus, neurophysiologic tests may be the best option for detecting early alterations [11–14].

Galvanic vestibular stimulation (GVS) applied to the mastoid bone triggers a motor response in posture-controlling muscles [15]. The electromyographic (EMG) response [i.e., the vestibular-evoked myogenic potential (VEMP)] can be generated using auditory or galvanic stimuli. EMG testing is safe, low-cost, and easily performed [15,16]. VEMP triggered by galvanic stimulation (galvanic-VEMP), elicits a more robust evoked response in the lower limb than that triggered by auditory stimulation (auditory-VEMP) [17]. Galvanic-VEMP assesses the central pathways of the vestibular system and provides information about the vestibulospinal tract extending from the cervical to lumbar spine [15,16,18]. Galvanic-VEMP testing has been used to investigate spinal cord function in cases of trauma, tumor, ischemia, and infection [15,16,19].

Bipolar and bilateral GVS is applied to the mastoid bones using a cathode on one side and an anode on the contralateral side [19]. The galvanic current induces an illusory perception of movement [19,20], which causes swaying of the body in the direction of the anode [21–23]. The central nervous system interprets the GVS response as a real body movement and activates compensatory postural reflex mechanisms [21]. Using surface electrodes, the galvanic-VEMP can be detected in the postural muscles, such as the triceps brachii, paraspinal, anterior tibial, soleus, and gastrocnemius [15,16,24]. The EMG response follows a characteristic normal pattern in healthy subjects that varies with the muscle that elicits the response and this has a relation with the level of the spine that the examiner aims at testing [24,25]. In the lower limb, the best EMG response is recorded from the gastrocnemius muscle [16]. The normal EMG wave pattern involves a short-latency (SL) response that begins at approximately 50 ms after the stimulus onset followed by a medium-latency (ML) response with opposite polarity that occurs at approximately 110 ms [20,25].

The aim of the present study was to use galvanic-VEMP to compare spinal cord function of HTLV-1-infected individuals in different levels of spinal damage that ranged from asymptomatic carrier to HAM.

## Methods

### Study design

For this cross-sectional comparative study, galvanic-VEMP was recorded from the lower limb of HTLV-1-asymptomatic carriers, individuals with possible HAM, individuals with HAM, and non-infected healthy individuals (controls).

### Ethical aspects

This research was conducted in accordance with the principles expressed in Declaration of Helsinki and it was approved by the Research Ethics Committee of the Federal University of Minas Gerais under number 266 and the Research Ethics Committee of the Hemominas Foundation under number 131. All participants provided voluntary written consent and declared that they were aware of the study procedures and their freedom to participate.

### Sample size

The sample size was calculated using G*Power software 3.1.9.2 (Heinrich-Heine-Universität Düsseldorf, Düsseldorf, Germany, 2007) to achieve a power of 80% and significance level of 5% based on the mean and standard deviation of the SL response of patients with HAM and healthy controls [26]. The final cohort of participants included 26 HTLV-1-asymptomatic carriers, 26 individuals with possible HAM, 25 individuals with HAM, and 45 individuals without HTLV-1 infection. Post hoc calculations of sample size indicated a power of 81% and α error of 3%.

### Participants of the study

The participants in this study were recruited from a cohort of former blood donors infected with HTLV-1 who have been followed by the Interdisciplinary HTLV Research Group (GIPH), since 1997, in Belo Horizonte, Minas Gerais State, Brazil [27, 28]. The main objective of GIPH is to study the natural history, clinical manifestations, epidemiology, and other aspects of HTLV infection [27, 28]. By the time of the present study, the cohort consisted of 637 HTLV-1-infected individuals and 232 non-infected, eligible blood donors (controls).

This present study was a transversal evaluation of 122 participants of the GIPH cohort. They were selected when they attended to the follow-up medical appointment scheduled by the GIPH and 160 individuals were invited to participate from February 2014 to June 2016. Twenty participants of the cohort refused to participate and 18 were excluded because of syphilis (n = 1), human immunodeficiency virus type 1 (HIV; n = 2), hepatitis B or C (n = 3), recurrent episodes of vertigo (n = 2), diagnosis of peripheral vestibular disease (n = 3), and inability to remain in a standing position (n = 7).

This study was composed by the controls [G1 (n = 45)], that were the HTLV-1-seronegative blood donors as assessed by enzyme-linked immune sorbent assay (ELISA)] and by the group of HTLV-1-infected participants (n = 77), that was the HTLV-1-seropositive blood donors as assessed by ELISA and confirmed by Western blot analysis (WB HTLV 2.4, Genelabs Diagnostics, Singapore) or by real-time polymerase chain reaction (PCR)] [29].

The HTLV-1 group was further divided into three subgroups according to neurological assessment. The HTLV-1-asymptomatic group [G2 (n = 26)] consisted of individuals without clinical symptoms according to Castro-Costa et al. criteria (S1 Table) [30] and scoring 0 on both the Expanded Disability Status Scale (EDSS) [31] and Osame’s Motor Disability Score (OMDS) [32]. The possible HAM group [G3 (n = 26)] consisted of individuals with HTLV-1 antibodies in serum and/or cerebrospinal fluid (CSF) confirmed by Western blot and/or positive PCR, having clinical symptoms according to Castro-Costa et al. criteria (S1 Table) [30], and neurological examination score of 1 to 2 on the EDSS [31] or OMDS [32]. The definite HAM group [G4 (n = 25)] consisted of individuals with presence of HTLV-1 antibodies in serum and CSF confirmed by Western blot and/or positive PCR, sufficient clinical symptoms of HAM according to Castro-Costa et al. criteria (S1 Table) [30], exclusion of other disorders that can resemble HAM, and neurological examination score of at least 2 on both EDSS and OMDS scales.

### Phases of the study

The participants were submitted to a clinical interview and neurological examination followed by galvanic-VEMP testing with the response recorded from the lower limb. Each participant was submitted to all the evaluations at the same day in the reference hospital of the Faculty of Medicine from UFMG, in Belo Horizonte, Minas Gerais State, Brazil. The evaluations were conducted by the same physicians, a specialist in infectious diseases, neuro-otologist and a neurologist.

The clinical interview considered the demographic variables (i.e., age, gender, and height), the medications in use, the complaint of dizziness, walking difficulties, history of vestibular disease and of stroke.

The neurological examination included the abdominal cutaneous, patellar tendons, and Achilles tendon reflexes, cranial nerves, sensitivity, muscle strength, gait, coordination, and abnormal movements. EDSS [31] and OMDS [32] evaluations were included in the neurological exam to classify HTLV-1-infected participants as asymptomatic carriers or individuals with possible HAM or definite HAM [S1 Table].

The GVS was applied on the mastoid bone and the VEMP response recorded from the lower limb. The GVS was characterized as a direct, monophasic, and rectangular current with an intensity of 2 mA and a duration of 400 ms (model EvP4/ATCPlus, Contronic, Ltd., Pelotas, Brazil). The bipolar current was applied on the mastoid processes using self-adhesive, circular surface electrodes (3 cm diameter; model CF3200, Valutrode, Axelgaard, Fallbrook, CA). For transmastoid binaural stimulation, the two current polarity settings used were cathode left and anode right (CLAR) and cathode right and anode left (CRAL). The stimulation polarity was controlled via a computer. For each test, four sets of 30 stimuli were applied and distributed, resulting in 30 responses recorded from the left lower limb (15 CLAR and 15 CRAL stimuli) and 30 responses recorded from the right lower limb (15 CLAR and 15 CRAL stimuli). This procedure was repeated for each leg to ensure data replication.

**Fig 1:**
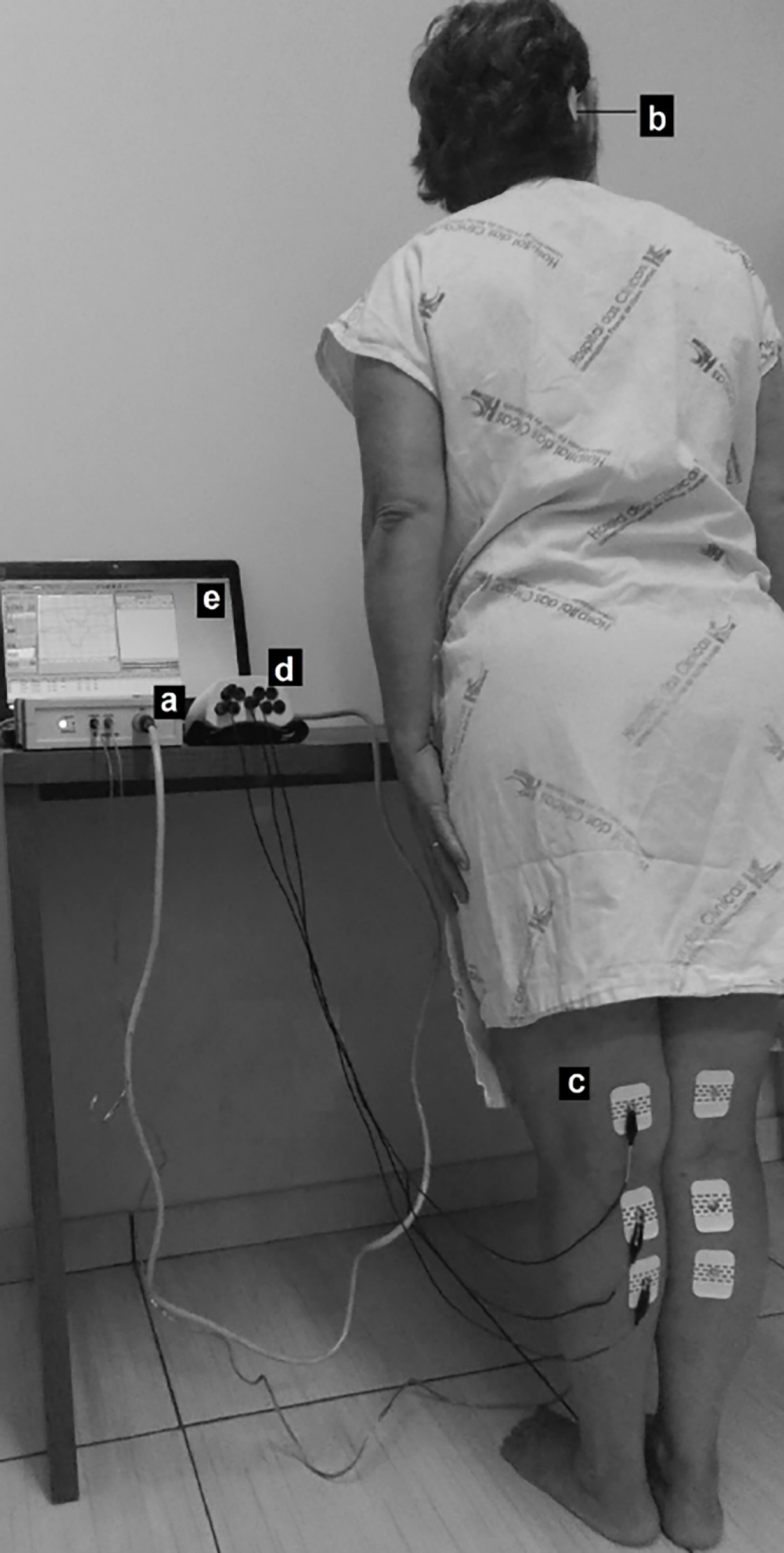
Vestibular-evoked myogenic potential triggered by galvanic vestibular stimulation (galvanic-VEMP) recording positions and equipment. It shows the right position of the participant to capture galvanic-VEMP; the equipment used for stimulus generation (a), the electrode positions for GVS (b), the electrode position to capture an electromyographic response on the gastrocnemius muscle (c), the equipment for processing the signal (d), and the computer for recording the responses (e).

During the procedure, the subjects stood barefoot on a flat surface with their eyes closed, feet close together, and bodies leaning forward to contract the gastrocnemius muscle [16,18,19]. To induce a stronger response, subjects were instructed to turn their heads approximately 90° to the side contralateral to the leg undergoing EMG response recording (Fig 1) [24].

The EMG response triggered by GVS was measured using self-adhesive electrodes (model 2223BRQ, 3M, Saint Paul, MN). A pair of recording electrodes was vertically placed 5 cm below the popliteal fossa on the medial head of the gastrocnemius muscle at approximately 5 cm between their centers. A reference electrode was placed on the back of the thigh at approximately 10 cm above the superior most recording electrode recording electrodes. The galvanic-VEMP was first measured in the left leg and then in the right leg. The tests were performed with a 2-min resting interval to avoid muscle fatigue (Fig 1).

The EMG signals were measured, corrected, filtered between 10 Hz and 1 kHz, and digitized at a sampling frequency of 5 kHz. Data were collected for 500 ms, starting 100 ms before GVS. EMG responses to 15 consecutive stimuli associated with each polarity configuration (i.e., CLAR and CRAL) were averaged to produce final traces. The galvanic-VEMP protocol is available at dx.doi.org/10.17504/protocols.io.nxbdfin.

### Data analysis

**Fig 2:**
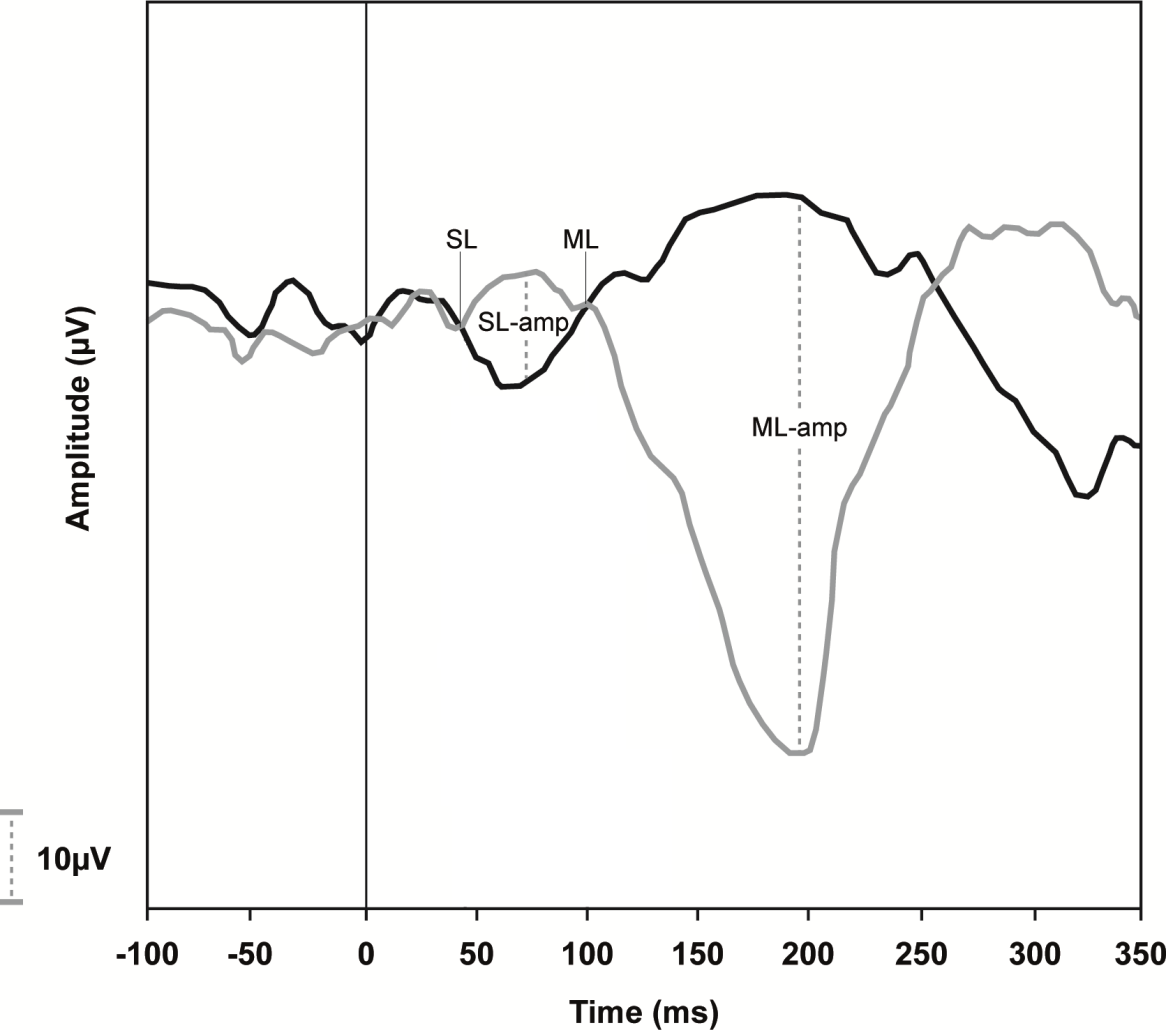
A normal vestibular-evoked myogenic potential triggered by galvanic vestibular stimulation (galvanic-VEMP) tracing. These recordings were obtained from an uninfected individual with his head rotated to the left and electromyographic (EMG) response recorded from the right gastrocnemius muscle. The black line indicates the trace recorded with the cathode and anode on the right and left side (CRAL), respectively, whereas the gray line indicates the opposite stimulation polarity (CLAR). SL, short-latency response (47 ms); ML, medium-latency response (100 ms); SL-amp, SL amplitude (12.5 μV); ML-amp, ML amplitude (62.5 μV).

Two independent examiners performed blind analyses of galvanic-VEMP traces. The traces were analyzed for the onset (in ms) of SL and ML responses as well as the amplitude (in μV) of SL (SL-amp) and ML (ML-amp) waves. Following the superimposition of traces with inverted polarity (i.e., CRAL and CLAR), the point where the traces diverged from the EMG baseline, which marked SL and ML onset, could be visualized and measured by a cursor. The first trace divergence, which occurred at approximately 50 ms, marked the onset of the SL response. Following this, the traces returned to baseline and then diverged again. The second trace divergence, which occurred at approximately 100 ms, marked the onset of the ML response (Fig 2). The amplitude of the SL and ML waves was measured as the maximal difference between the positive and negative traces of each wave (Fig 2).

Data were analyzed using EpiData 3.0 (EpiData Data Entry, Data Management, and Basic Statistical Analysis System, The EpiData Association, Odense, Denmark) and SPSS 15.0 (SPSS, Inc., Chicago, IL) software. The Shapiro-Wilk test was used to analyze the asymmetric distributions of all continuous variables. Among them, the age showed a normal distribution, so that it was presented as media (± standard deviation); the height, SL onset, ML onset, SL-amp, and ML-amp showed non-normal distribution, so that they were presented as median (Quartil Q1/Quartil Q3).

To assess possible confounding variables, a multiple linear regression analysis by backward elimination was performed. The independent variables were height, age, sex, and group. The dependent variables were SL onset, ML onset, SL-amp, and ML-amp.

The Spearman correlation coefficient was calculated to measure the concordance degree between the two independent examiners. The correlation was classified as weak (r = 0–0.40), moderate (r = 0.41–0.70), or strong (r = 0.71–1).

To determine whether the galvanic-VEMP response was normal or altered, we constructed the receiver operating characteristic (ROC) curves and established SL and ML cut-off values based on sensitivity and specificity analyses. Neurological examination (i.e., normal or altered) were considered as the gold standard.

Between-group comparisons of continuous variables were performed using a Kruskal-Wallis test with Bonferroni correction. Between-group comparisons of categorical variables were performed using a Chi-square or Fisher’s exact test. A significance level was defined as 5%.

## Results

### Characteristics of groups with and without HTLV-1 infection

The uninfected control group (G1) consisted of 45 HTLV-1-seronegative subjects. The HTLV-1-seropositive group (n = 77) was divided into three subgroups, including 26 asymptomatic individuals (G2), 26 individuals with possible HAM (G3), and 25 individuals with HAM (G4).

The general characteristics of the participants are shown in Table 1. The groups were similar regarding age, height, and sex. The main results of the clinical and neurological evaluations were included in the S2 Table.

**Table 1:** General characteristics of human T-cell lymphotropic virus type 1 (HTLV-1)-infected and -uninfected groups (n = 122).

Variables	G1 (n = 45)	G2 (n = 26)	G3 (n = 26)	G4 (n = 25)	p-value
**Age (years)**	52±10	52±11	56±9	55±9	0.281^a^
**Height (m)**	1.64 (1.60/1.70)	1.58 (1.55/1.70)	1.59 (1.56/1.66)	1.62 (1.56/1.72)	0.207^b^
**Women**	28 [62]	15 [58]	17 [65]	18 [72]	0.747^c^

G1, uninfected control group; G2, HTLV-1-asymptomatic group; G3, possible HTLV-1-associated myelopathy (HAM) group; G4, HAM group; n, number of participants. Data are expressed as mean ± standard deviation for continuous variables with normal distribution, median (Quartil Q1/Quartil Q3) for continuous variables with non-normal distribution, and as absolute number [percentage] for categorical variables.

^a^Analysis of variance (ANOVA)

^b^Kruskal-Wallis test

^c^chi-square test.

### Potential confounders

According to multivariate analysis, the potential confounders age, sex, height, and weight did not influence the variables of interest. The results indicated that the group type was associated with changes in SL (β = 0.62, p<0.001) and ML (β = 0.58, p<0.001) responses.

The blind analyses of galvanic-VEMP traces performed by the two examiners were strongly correlated for SL (r = 0.713, p<0.001) and ML (r = 0.812, p<0.001) and moderately correlated for SL-amp (r = 0.521, p<0.001) and ML-amp (r = 0.62, p<0.001).

For each group, there was no significant difference between the EMG responses of the right and left legs (p>0.05). Thus, the group comparisons were performed using SL and ML measurements from either the right or left leg. To increase the sensitivity of the analysis, for all participants, we analyzed the measurements taken from the leg presenting higher latencies.

### SL and ML responses in HTLV-1-infected and -uninfected groups

The latency and amplitude of SL and ML responses are shown in Table 2. SL and ML response latencies were different between groups, where as SL-amp and ML-amp were similar between groups.

**Table 2:** Comparative analysis of the latency and amplitude of short-latency (SL) and medium-latency (ML) responses between human T-cell lymphotropic virus type 1 (HTLV-1)-infected and -uninfected groups.

Variables	G1 (n = 45)	G2 (n = 26)	G3 (n = 26^a^)	G4 (n = 25^b^)	p-value^c^	Post hoc analysis^d^
**SL (ms)**	54 (50/55)	63 (59/69)	67 (58/71)	70 (65/72)	<0.001	G1<G2; G1<G3; G1<G4; G2<G4.
**ML (ms)**	111 (103/114)	118 (106/124)	124 (121/132)	144 (127/154)	<0.001	G1<G2; G1<G3; G1<G4; G2<G3; G2<G4; G3<G4.
**SL-amp (**μ**V)**	16 (11/23)	14 (11/24)	13 (10/24)	16 (9/26)	0.970	_
**ML-amp (**μ**V)**	37 (28/62)	35 (27/50)	36 (20/54)	33 (14/55)	0.093	_

G1, uninfected control group; G2, HTLV-1-asymptomatic group; G3, possible HTLV-1-associated myelopathy (HAM) group; G4, HAM group; SL, short-latency response; ML, medium-latency response; SL-amp, SL amplitude; ML-amp, ML amplitude; n, number of participants; ms, milliseconds; μV, microvolt. Data are expressed as median (Quartil Q1/Quartil Q3) for continuous variables with non-normal distribution.

^a^For SL data analysis, three cases with absent response were excluded.

^b^For SL and ML data analysis, 15 and 11 cases with absent responses were excluded, respectively.

^c^Kruskal-Wallis test.

^d^Post hoc Bonferroni-corrected p-values for specific comparisons (p<0.008).

SL responses were significantly delayed in the HTLV-1-asymptomatic, possible HAM, and HAM groups compared to the control group SL response. ML responses were longer in the possible HAM and HAM groups compared to those of the HTLV-1-asymptomatic and control groups. The longest latencies were found in patients with the most severe neurological impairment.

There was a significant correlation between EDSS and ML responses in possible HAM and HAM groups, showing that there was a parallel between the clinical profile (evaluated by EDSS or OMDS) and ML latencies. The correlation between EDSS and ML was strong (p<0.001 and r = 0.707) and between OMDS and ML was moderate (p<0.001 and r = 0.642) (S1 Fig).

### Types of galvanic-VEMP responses in HTLV-1-infected groups

**Fig 3:**
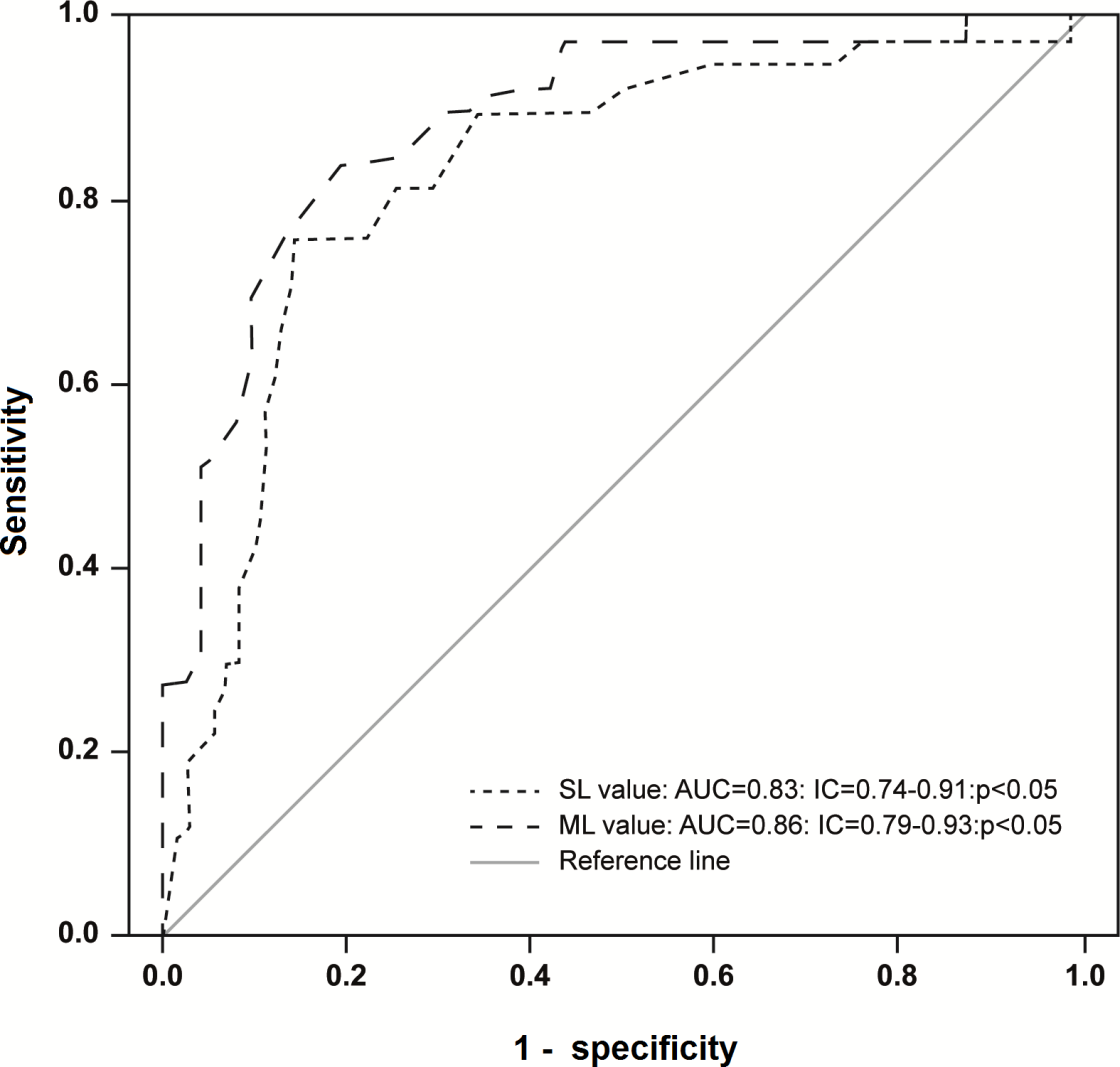
Short-latency (SL) and medium-latency (ML) response receiver operating characteristic (ROC) curves for detecting spinal cord injury in patients infected with human T-cell lymphotropic virus type 1 (HTLV-1). AUC, area under the curve; IC, confidence interval.

We classified the galvanic-VEMP response as normal or altered. An altered galvanic-VEMP response occurred when the SL or the ML wave from at least one leg was delayed or absent. Using neurological examination as the gold standard, we constructed the receiver operating characteristic (ROC) curves to evaluate delayed SL and ML latencies. SL and ML responses presented good areas under the curve (AUC; Fig 3).

**Fig 4:**
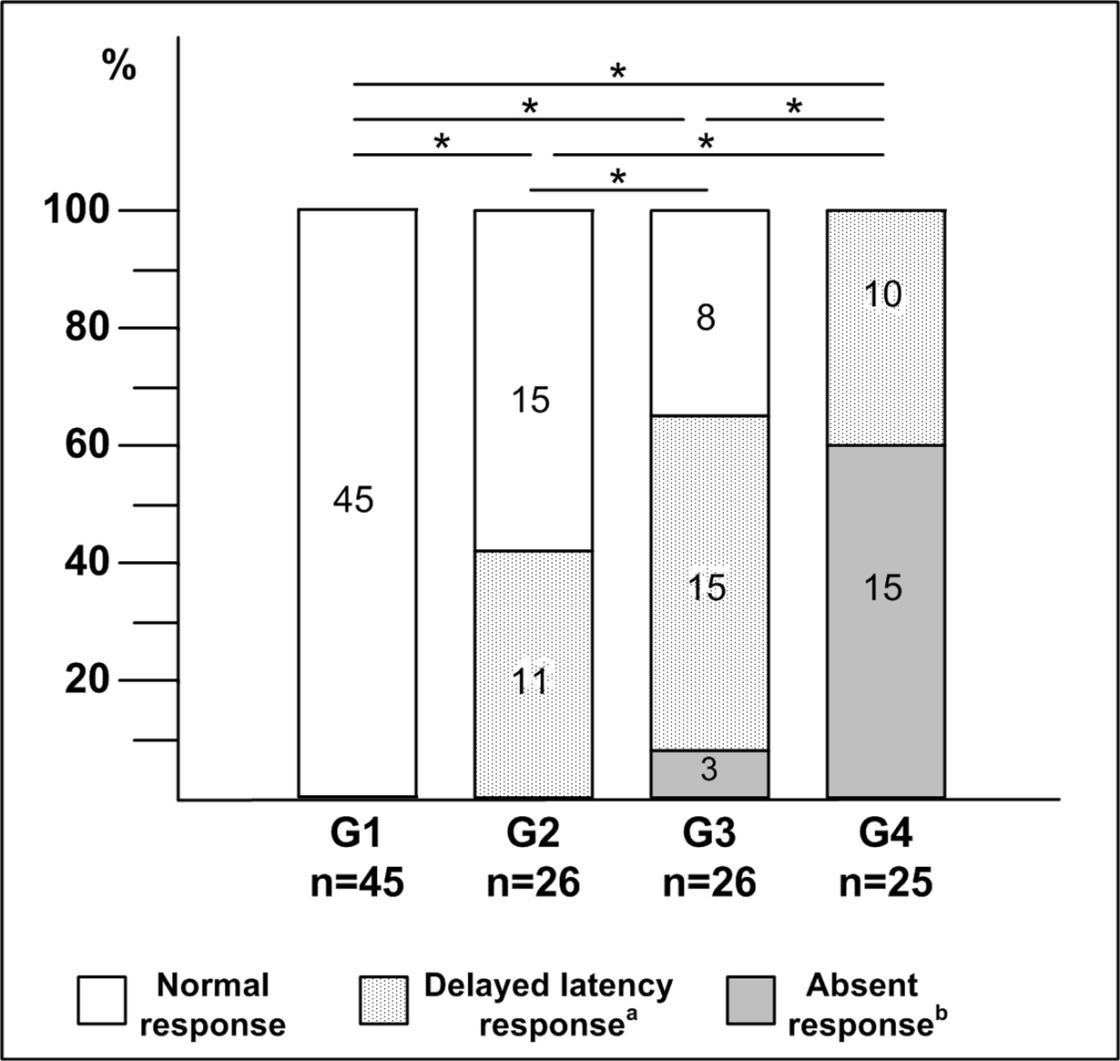
Frequency comparisons between normal and altered vestibular-evoked myogenic potential triggered by galvanic vestibular stimulation (galvanic-VEMP) for each group. The type of response (normal, delayed latency, or absent) and its frequency (%) is shown for each group. G1, uninfected control group; G2, human T-cell lymphotropic virus type 1 (HTLV-1)-asymptomatic group; G3, possible HTLV-1-associated myelopathy (HAM) group; G4, HAM group; n, number of participants. ^a^Delayed latency of short-latency (SL), medium-latency (ML), or both responses. ^b^Absent SL, ML, or both responses. *p<0.001, chi-square or Fisher’s exact test.

The best cut-off values for SL and ML responses were 64 ms (sensitivity: 76%, specificity: 86%) and 122 ms (sensitivity: 79%, specificity: 85%), respectively. To determine if the SL or ML response from at least one leg was absent, the galvanic-VEMP was performed twice. When SL or ML responses were absent, the galvanic-VEMP was repeated three times. Fig 4 shows normal and altered galvanic-VEMP frequency (percentage) and indicates the types of alterations found in each group. The galvanic-VEMP was more frequently altered in the HAM group compared to the possible HAM, HTLV-1-asymptomatic, and control groups (p<0.001).

**Fig 5:**
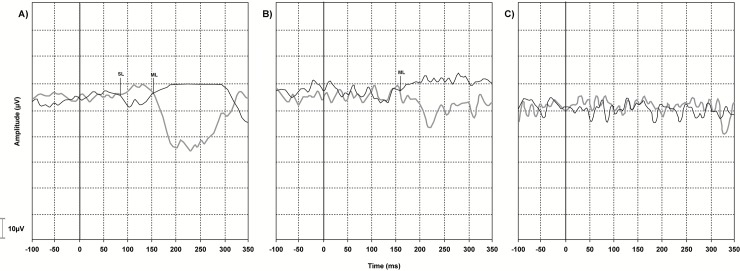
Vestibular-evoked myogenic potential triggered by galvanic vestibular stimulation (galvanic-VEMP) tracings. (A) Delayed short-latency (SL) and medium-latency (ML) waves in an individual with asymptomatic human T-cell lymphotropic virus type 1 (HTLV-1) infection. (B) Absent SL response with delayed ML response latency in an individual with possible HTLV-1-associated myelopathy (HAM). (C) Absent SL and ML responses in an individual with HAM. These recordings were obtained from subjects with their heads rotated to the left and electromyographic (EMG) responses recorded from the right gastrocnemius muscle. The black line indicates the trace recorded with the cathode and anode on the right and left side respectively, whereas the gray line indicates the opposite stimulation polarity. SL, short-latency response onset; ML, medium-latency response onset.

In the HTLV-1-asymptomatic group, the most frequent type of altered response was SL delayed latency (Fig 5A). In the possible HAM group, predominated a delay in both SL and ML latencies (Fig 5B). In the HAM group, the most common altered response was the absence of SL and ML, which means no electrophysiological response (Fig 5C). To sum up, we found that the worse the galvanic-VEMP response, the more severe the myelopathy was.

## Discussion

In the present study, galvanic-VEMP disclosed an altered EMG response of the inferior limb in HTLV-1-infected individuals that otherwise were considered asymptomatic carrier. For the SL and ML components of the EMG wave, a greater latency delay was associated with a greater motor impairment.

VEMP triggered by acoustic stimulation has been used to evaluate the brainstem and cervical spine [33,34], including the investigation of patients with HTLV-1 infection [13,14,34]. Interestingly, the pattern of alteration found in our study using galvanic stimulation was similar to a previous one using acoustic stimulation: the worst EMG wave in cervical VEMP was associated with the more severe myelopathy [14]. However, the acoustic stimulus does not differentiate between central and peripheral vestibular disorders. Galvanic-VEMP has the advantages of directly stimulating the vestibular nucleus and providing stronger stimulus [17]. Thus, galvanic-VEMP is more appropriate to evaluate the central vestibular pathway and the spinal cord disorders [15,16,35]. Taking into consideration that galvanic-VEMP stimulates the entire spinal cord and that HAM is a motor myelopathy that starts at the thoracic spine and slowly progresses upward in the spine, the galvanic-VEMP with the response recorded from the inferior members may be an interesting tool for the early diagnosis of HAM [30,36] and for the following-up of these patients in case of future therapeutic strategies [37].

In S3 Table, we showed the comparison between the results using the VEMP recorded from the lower limb muscle (galvanic-VEMP) and VEMP recorded from the cervical muscle (cervical-VEMP). The analysis in terms of the spinal level of electrophysiological response allows the conclusion that a normal VEMP response in the cervical muscle with an altered VEMP response in the lower limb muscle would indicate an injury at the lumbar-thoracic level with a probably yet normal cervical spine. Considering that HAM onset occurs more commonly at the lumbar thoracic level [3,4,5], galvanic-VEMP response in the lower limb muscle represents a better tool for the early diagnosis of HAM comparing to VEMP response from the cervical muscle.

The altered patterns of galvanic-VEMP response included delayed latency or EMG wave absence, which indicated damage to the vestibular pathways [16,18,26]. In a previous study involving eight patients with spinal cord injury of varying etiology, the ML and SL components recorded in these patients had prolonged latencies compared to eight healthy controls (p<0.005) [18]. In another study of 21 patients with spinal cord injury, galvanic-VEMP latency correlated with the severity of spinal cord impairment (r_s_ = -0.57; p<0.01) [15]. In our study, similar findings were observed in 122 individuals, including 45 healthy controls and 77 HTLV-1-infected individuals in different phases of the disease. A previous study had already shown the lower limb galvanic-VEMP latency delay in HTLV-1-asymptomatic carriers comparing to controls [26]. To better document the extent of electrophysiological damage and to better compare to the response of the HAM patients, we included individuals with possible HAM according to the criteria of Castro-Costa et al. (S1 Table) [30]. These individuals presented an altered pattern of response that was intermediate between those observed in the group of asymptomatic carriers and with definite HAM. The inclusion of patients with possible HAM was important to clarify a possible progression of the EMG alterations from the asymptomatic profile to the defined myelopathy. It is possible that HTLV-1-asymptomatic carriers with altered galvanic-VEMP are more likely to develop HAM soon as opposed to HTLV-1-asymptomatic carriers with normal galvanic-VEMP. Prospective studies are necessary to confirm this hypothesis.

To analyze the electrophysiological tests, it is necessary to categorize them as normal or altered. Thus, we established SL and ML cut-off values based on our results and published data [16,17,20,24,25]. Using neurological examination as the gold standard, we found that the most reliable cut-off values in the ROC curves (Fig 3) for SL and ML were 64 ms (sensitivity: 76%, specificity: 86%) and 122 ms (sensitivity: 79%, specificity: 85%), respectively.

Categorical analysis of the galvanic-VEMP responses revealed a gradient of altered responses from the HTLV-1-asymptomatic carrier to HAM. The control group presented normal galvanic-VEMP responses, whereas 42% of HTLV-1-asymptomatic carriers, 69% of the individuals with possible HAM, and 100% of the individuals with HAM had altered response (Fig 4). The tracings shown in Fig 5 demonstrate progressive differences in the pattern of altered galvanic-VEMP responses. The SL was the first wave to alter, being followed by alteration in ML wave. Consecutively, the SL wave disappeared, being followed by the disappearance of ML wave. The absence of the galvanic-VEMP response has been shown to be common in patients with spinal cord injury [15,16,18,26].

### HTLV-1 infection affects the reticulospinal and vestibulospinal tracts

GVS seems to act primarily on the presynaptic regions of vestibular afferent nerves within otoliths and semicircular canals [19]. Cathodal galvanic stimuli depolarize, whereas anodal stimuli hyperpolarize afferent vestibular fibers [38,39].

The SL and ML responses seem to have different origins and pathways [19]. The SL component of the galvanic-VEMP wave presumably originates from the otoliths and the stimulus follows by the reticulospinal tract, whereas the polysynaptic ML component seems to originate from the semicircular canals and follows by the vestibulospinal tract [24,38,34]. Thus, SL and ML alterations indicate respectively reticulospinal and vestibulospinal tract dysfunction. Actually, SL and ML responses have a certain degree of independence [40]. For example, visual input can nearly abolish the ML response without affecting the SL curve. Moreover, the ML response changes when posture is supported by one finger or with reduced cutaneous sensory input from the feet [41]. However, the SL response is stable in these situations, but is more difficult to measure than the ML response [40].

In our study, the amplitude of the ML response was larger than that of the SL response (Fig 2), which clearly explains why the analyses performed by the two independent examiners were more strongly correlated for the ML response (r = 0.812) than for the SL response (r = 0.713).

### Galvanic-VEMP can be useful for monitoring HTLV-1-associated neurological alterations

We have observed that a pattern of galvanic-VEMP alteration starts with the latency delay in HTLV-1-asymptomatic carriers that is followed by the absence of the EMG response in individuals with possible HAM and definite HAM [13,14]. It is taken for granted that EMG alteration occurs before the clinical manifestation. Interestingly, HAM patients frequently experience dizziness before any other clinical manifestation [14,42]. As the reticulospinal and vestibulospinal tracts are tested by VEMP, the present results indicate that these tracts are compromised very early in HAM.

Researchers have been using clinical, biological and electrophysiological evaluations to determine possible biomarkers for HAM. The HTLV-1 provirus load and the immunological inflammatory profile have been studied under this concept [28, 43, 44]. In addition, host traits, age, inflammatory mediators, and gender has also been pointed as factors that can exerted an influence on HAM development [45]. Electrophysiological alterations in HTLV-1, as shown in the present article, may complement the battery of possible biomarkers of HAM.

Electrophysiological exams can help to determine the damage extension of the central nervous system in patients with HAM [4,12,15,46,47,48]. Previous studies have shown that the central motor conduction time (CMCT) is significantly prolonged or altered in HAM [47,48]. These results reflect the extension of the lesions in the anterior horn cells caused by HAM [47,48].

### Potential confounding factors

We examined the latency and amplitude of the galvanic-VEMP responses. SL and ML latencies were different between groups (Table 2). In contrast, SL and ML amplitudes were similar between groups. Several studies have shown that VEMP amplitude can be modified by the stimulus parameters, environment, age, height, gender, and spinal cord damage [15,19,21,22,49]. Galvanic-VEMP amplitude decreases and ML is more delayed with aging [15,35]. The ML is more prolonged in women than in men [22]. Moreover, VEMP latency was also found to correlate with height [35]. In our study, we controlled the potential confounding variables such as height, gender and age.

### Limitations

We did not include patients with HAM who could not stand, because galvanic-VEMP responses were recorded from the muscles engaged in the maintenance of a standing posture. It is possible to record VEMP from erectors spinae muscles using GVS in sitting patients [15]. If we had tested the erectors spinae muscles, we could have attained better precision in the detection of the level of neurological damage; however, this information would not be relevant since HAM was already defined and the target population of the present study was the asymptomatic carrier aiming at disclosing the subclinical impairment.

Another limitation was the lack of a battery of electrophysiological tests (nerve conduction study, somatosensory evoked potential, and motor evoked potential by magnetic stimulation) to exclude other causes of neuropathies. All the HTLV-1-infected individuals followed by GIPH are submitted to neurological examination every two year to ruled out other neurological diseases [S1 Table].

## Conclusions

The worse spinal cord damage in HTLV-1 infection, the worse galvanic-VEMP response, characterizing a gradient that ranged from a delayed latency in the HTLV-1-asymptomatic carriers and individuals with possible HAM to an absent response in individuals with confirmed HAM. Galvanic-VEMP is a promising tool for improving the early diagnosis of HAM and might be used for the following-up of patients under future therapeutic strategies.

## Supporting information

S1 Table Diagnostic criteria of human T-cell lymphotropic virus type 1 (HTLV-1)-associated myelopathy (HAM). (PDF)

S2 Table Clinical aspects of the human T-cell lymphotropic virus type 1 (HTLV-1)-infected (n = 77) and -uninfected individuals (n = 45). G1, uninfected control group; G2, HTLV-1-asymptomatic group; G3, possible HTLV-1-associated myelopathy (HAM) group; G4, HAM group; n, number of participants; EDSS, Expanded Disability Status Scale; OMDS, Osame’s Motor Disability Score; Data are expressed as median (Quartil Q1/Quartil Q3) for continuous variables with non-normal distribution, and as absolute number [percentage] for categorical variables.
(PDF)

S3 Table Comparison between the VEMP recorded from the lower limb muscle and the VEMP recorded from the cervical muscle in individuals with possible HAM (n = 17) and with definite HAM (n = 18). Data are expressed as absolute number (percentage); n, number of participants with performed both VEMP recorded from the lower limb muscle and VEMP recorded from the cervical muscle.
(PDF)

S1 Fig Galvanic-VEMP medium-latency response related to symptoms in possible HAM and HAM groups. **(A) EDSS values *versus* galvanic-VEMP medium-latency response, (B) OMDS values *versus* galvanic-VEMP medium-latency response.** ML, medium-latency response; EDSS, Expanded Disability Status Scale; OMDS, Osame’s Motor Disability Score; p, probability of significance; r, Spearman correlation coefficient.
(TIF)
