# Supplementary material for: Vestibular-evoked myogenic potential triggered by galvanic vestibular stimulation may reveal subclinical alterations in human T-cell lymphotropic virus type 1-associated myelopathy
Source: PLoS One. 2018 Jul 12;13(7):e0200536. doi: 10.1371/journal.pone.0200536 (PMC6042765; doi:10.1371/journal.pone.0200536)
Supplement: S1 Table — (PDF) [file pone.0200536.s001.pdf]

S1 Table. Diagnostic criteria of human T-cell lymphotropic virus type 1 (HTLV-1)-associated myelopathy (HAM)[a]

**Definite:**
1. A non-remitting progressive spastic paraparesis with sufficiently impaired gait to be perceived by the patient. Sensory symptoms or signs may or may not be present. When present, they remain subtle and without a clear-cut sensory level. Urinary and anal sphincter signs or symptoms may or may not be present.
2. Presence of HTLV-1 antibodies in serum and cerebrospinal fluid (CSF) confirmed by Western blot and/or a positive real-time polymerase chain reaction (PCR) for HTLV-1 in blood and/or CSF.
3. Exclusion of other disorders that can resemble HAM[b].
**Probable:**
1. Mono-symptomatic presentation: spasticity or hyperreflexia in the lower limbs or isolated Babinski sign with or without subtle sensory signs or symptoms, or neurogenic bladder only confirmed by urodynamic tests.
2. Presence of HTLV-1 antibodies in serum and/or CSF confirmed by Western blot and/or a positive PCR for HTLV-1 in blood and/or CSF.
3. Exclusion of other disorders that can resemble HAM[b].
**Possible:**
1. Complete or incomplete clinical presentation.
2. Presence of HTLV-1 antibodies in serum and/or CSF confirmed by Western blot and/or a positive PCR for HTLV-1 inblood and/or CSF.
3. Disorders that can resemble HAM have not been excluded[b].

[a]Castro-costa CMDE, Araújo AQC, Barreto MM, Takayanagui OM, Sohler MP, Silva ELMDA, et al. Proposal for diagnostic criteria of tropical spastic paraparesis/HTLV-1- associated myelopathy (HAM/TSP). AIDS Res Hum Retroviruses. 2006;22:931–935. Doi: 10.1089/aid.2006.22.931.

[b]To minimize a misdiagnosis with disorders that can resemble HAM, the following conditions should be excluded by apropriate laboratory and clinical evaluation: multiple sclerosis; carcinomatous meningitis; familial spastic paraparesis; transverse myelitis; primary lateral sclerosis; paraneoplastic syndromes; syringomyelia; Lyme disease; B12 and folate deficiency; Behçet's disease; neurosyphilis; neurotuberculosis; sarcoidosis; HIV-associated vacuolar myelopathy; collagen vascular diseases; autoimmune myelopathies; Sjogren's syndrome; toxic myelopathies; amyotrophic lateral sclerosis; fungal myelopathy; spinal arteriovenous fistula; hepatic myelopathy; spinal cord compression (spinal tumor, cervical spondylosis, brain parasagittal tumor); endemic regional myelopathies with similar clinical manifestations (including schistosomiasis and neurocysticercosis).
