# Supplementary material for: Vestibular-evoked myogenic potential triggered by galvanic vestibular stimulation may reveal subclinical alterations in human T-cell lymphotropic virus type 1-associated myelopathy
Source: PLoS One. 2018 Jul 12;13(7):e0200536. doi: 10.1371/journal.pone.0200536 (PMC6042765; doi:10.1371/journal.pone.0200536)
Supplement: S2 Table — G1, uninfected control group; G2, HTLV-1-asymptomatic group; G3, possible HTLV-1-associated myelopathy (HAM) group; G4, HAM group; n, number of participants; EDSS, Expanded Disability Status Scale; OMDS, Osame’s Motor Disability Score; Data are expressed as median (Quartil Q1/Quartil Q3) for continuous variables with non-normal distribution, and as absolute number [percentage] for categorical variables. (PDF) [file pone.0200536.s002.pdf]

S2 Table. Clinical aspects of the human T-cell lymphotropic virus type 1 (HTLV-1) infected (n=77) and -uninfected individuals (n=45)

Variables	G1	G2	G3	G4
	(n=45)	(n= 26)	(n= 26)	(n= 25)
**Dry eye**	5 [11]	4 [15]	6 [23]	14 [56]
**Constipation**	3 [6]	5 [19]	9 [35]	19 [76]
**Back pain**	4 [9]	4 [15]	6 [23]	17 [68]
**Urinary symptoms**	0	0	4 [15]	19 [76]
**Sensory symptoms**	0	0	9 [35]	12 [48]
**Babinski sign**	0	0	7 [27]	17 [68]
**Reduced strength**	0	0	3 [12]	14 [56]
**Gait disturbance**	0	0	14 [54]	25 [100]
**EDSS**	0	0	2 (1/2)	6 (5/7)
**OMDS**	0	0	1 (0/1)	5 (4/5)

G1, uninfected control group; G2, HTLV-1-asymptomatic group; G3, possible HTLV-1-associated myelopathy (HAM) group; G4, HAM group; n, number of participants; EDSS, Expanded Disability Status Scale; OMDS, Osame's Motor Disability Score; Data are expressed as median (Quartil Q1/Quartil Q3) for continuous variables with non-normal distribution, and as absolute number [percentage] for categorical variables.
