# Supplementary material for: Vestibular-evoked myogenic potential triggered by galvanic vestibular stimulation may reveal subclinical alterations in human T-cell lymphotropic virus type 1-associated myelopathy
Source: PLoS One. 2018 Jul 12;13(7):e0200536. doi: 10.1371/journal.pone.0200536 (PMC6042765; doi:10.1371/journal.pone.0200536)
Supplement: S3 Table — Data are expressed as absolute number (percentage); n, number of participants with performed both VEMP recorded from the lower limb muscle and VEMP recorded from the cervical muscle. (PDF) [file pone.0200536.s003.pdf]

S3 Table. Comparison between the VEMP recorded from the lower limb muscle and the VEMP recorded from the cervical muscle in individuals with possible HAM (n=17) and with definite HAM (n=18).

Group	Response	VEMP recorded from the lower limb muscle	VEMP recorded from the cervical muscle
G3 - Possible HAM (n=17)	Normal	6 (35)	13 (76)
	Delayed latency	10 (59)	4 (24)
	Absent	1 (6)	0
G4 – Definite HAM (n=18)	Normal	0	10 (55)
	Delayed latency	7 (39)	5 (28)
	Absent	11 (61)	3 (17)

Data are expressed as absolute number (percentage); n, number of participants with performed both VEMP recorded from the lower limb muscle and VEMP recorded from the cervical muscle.
