# Supplementary figures and images for: Vestibular-evoked myogenic potential triggered by galvanic vestibular stimulation may reveal subclinical alterations in human T-cell lymphotropic virus type 1-associated myelopathy
Source: PLoS One. 2018 Jul 12;13(7):e0200536. doi: 10.1371/journal.pone.0200536 (PMC6042765; doi:10.1371/journal.pone.0200536)

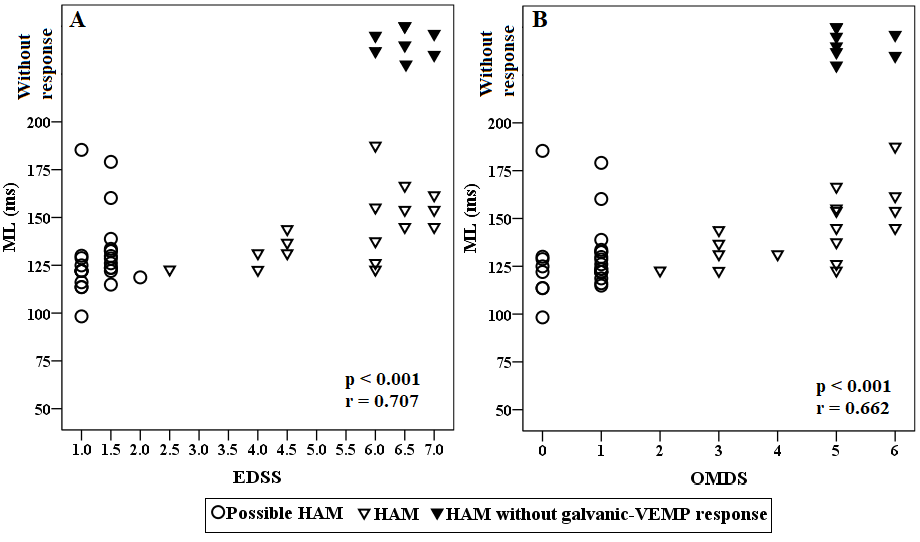

Supplement: S1 Fig — (A) EDSS values versus galvanic-VEMP medium-latency response, (B) OMDS values versus galvanic-VEMP medium-latency response. ML, medium-latency response; EDSS, Expanded Disability Status Scale; OMDS, Osame’s Motor Disability Score; p, probability of significance; r, Spearman correlation coefficient. (TIF) [file pone.0200536.s004.tif]
